# Supplementary material for: The Impact of Limited Previous Motor Experience on Action Possibility Judgments in People with Spinal Muscle Atrophy
Source: Brain Sci. 2023 Aug 29;13(9):1256. doi: 10.3390/brainsci13091256 (PMC10526987; doi:10.3390/brainsci13091256)
Supplement: Supplementary file 1 [file brainsci-13-01256-s001.zip › brainsci-2576062-supplementary.pdf]

Supplementary File

Investigating the relationship between sensorimotor characteristics and predicted movement times

Start of Block: Informed Consent

Welcome to the research study!

We are interested in understanding [STUDY TOPIC]. For this study, you will be presented with information relevant to [STUDY TOPIC]. Then, you will be asked to answer some questions about it. Your responses will be kept completely confidential.

The study should take you around [SURVEY DURATION IN MINUTES] to complete. You will receive [INCENTIVE] for your participation. Your participation in this research is voluntary. You have the right to withdraw at any point during the study. The Principal Investigator of this study can be contacted at [NAME/ EMAIL ADDRESS].

By clicking the button below, you acknowledge:
Your participation in the study is voluntary. You are 18 years of age. You are aware that you may choose to terminate your participation at any time for any reason.

- I consent, begin the study
- I do not consent, I do not wish to participate

What type of device are you using (Computer, Tablet, Phone?). If you can please specify the model (e.g., 2013 Lenovo Thinkpad)

____

What is the estimated screen size of the device (e.g., 13 inches) ?

____

What is your Age in Years (e.g., 25)

____

What is your sex (e.g., Female, Male, prefer not to disclose)?

____

Please indicate which hand you would use for the following activities:

	Right	Left	Both	None
Using an assistive device (e.g. wheel chair controller).	o	o	o	o
Pointing to a location	o	o	o	o
Using a mouse	o	o	o	o
Holding keys/objects	o	o	o	o
Using a writing device or tablet	o	o	o	o

Please view the following video for the instructions

○ I have viewed the instructions

Page Break

Please note your participant ID: **P${rand://int/1:9}${rand://int/1:9}${rand://int/1:9}${rand://int/1:9}**
You will have to enter it to complete the experiment

${date://CurrentDate/SL} ${date://CurrentTime/TL}

End of Block: Informed Consent

Table S1. *An example participants' scoring system on the adapted Spinal Muscular Atrophy Health Index*

How much does the following impact your life now (Score)	I don't experience this (0)	I experience this but it does not affect my life (1)	It affects my life a little (2)	It affects my life moderately (3)	It affects my life very much (4)	It affects my life severely (5)
**Difficulty lifting objects**						X
**Arm weakness**						X
**Problems reaching things over your head**						X
**Difficulty propping yourself up with your arms**						X
**Decreased arm and shoulder range of motion**						X
**Shoulder weakness**						X
**Hand weakness**						X
**Difficulty doing things with your hands**						X
**Impaired ability to open doors or drawers (Heaviness or grabbing the door?)**						X
**Difficulty washing your hair**						X
**Weak hand grasp**						X
**Difficulty picking things up with your fingers**						X
**Difficulty maintaining personal cleanliness**						X
**Dropping objects with your hands**						X
**Difficulty holding a pen or pencil**						X
**Difficulty moving small objects with your hands**						X
**Which of the following best describes how you get around? Ex: Walk**	I use a wheelchair. (5)					

**independently, cane, walker, wheelchair, motorized scooter…**	
**Overall SMAHI score**	(5 x 16) /16 = 5

Note. Adapted from *The Spinal Muscular Atrophy Health Index* (Heatwole et al., 2015).
